# Epidemiology of Dermatophytes Isolated from Clinical Samples in a Hospital in Eastern Saudi Arabia: A 20-Year Survey

**DOI:** 10.1007/s44197-021-00005-5

**Published:** 2021-09-16

**Authors:** Bashayer Ali Alshehri, Aisha M. Alamri, Ali A. Rabaan, Jaffar A. Al-Tawfiq

**Affiliations:** 1 Department of Laboratory Services, Johns Hopkins Aramco Healthcare, Dhahran, Kingdom of Saudi Arabia; 2 Department of Clinical Laboratory Sciences, College of Applied Medical Sciences, Imam Abdulrahman Bin Faisal University, Dammam, Kingdom of Saudi Arabia; 3 Molecular Diagnostics Laboratory, Johns Hopkins Aramco Healthcare, Dhahran, Kingdom of Saudi Arabia; 4 Department of Public Health and Nutrition, The University of Haripur, Haripur, Pakistan; 5 Infectious Disease Unit, Specialty Internal Medicine, Johns Hopkins Aramco Healthcare, Dhahran, Kingdom of Saudi Arabia; 6 Infectious Diseases Division, Department of Medicine , Indiana University School of Medicine, Indianapolis, IN USA; 7 Infectious Diseases Division, Department of Medicine , Johns Hopkins University School of Medicine, Baltimore, MD USA; 8 Dhahran Health Center, Kingdom of Saudi Arabia, Johns Hopkins Aramco Healthcare, Room D-0032, Building 61, P.O. Box 76, Dhahran, 31311 Kingdom of Saudi Arabia

**Keywords:** *Microsporum*, *Trichophyton*, *Epidermophyton*, Tinea, Dermatophytosis, Dermatophytes

## Abstract

**Background:**

Dermatophytes are group of fungi that cause superficial infections via enzymes that degrade keratin in human skin. Several factors, including climate, gender, age, lifestyle, human migration, cultural habits, and socioeconomic status influence the prevalence of dermatophyte infections. We analyzed the prevalence of dermatophyte isolates in a hospital in Eastern Saudi Arabia from 2000 to 2019.

**Methods:**

The data on fungal cultures were obtained from the Laboratory Information System of the Mycology Laboratories at Johns Hopkins Aramco Healthcare, and were used for the analysis. Fungal isolates were examined microscopically for the presence of specialized hyphal structures and conidia. The Vitek^®^ MS microbial identification system (biomerieux) was used if the culture type was not identified microscopically.

**Results:**

Among the 10,021 samples analyzed, 3040 (30.33%) were positive for fungi and only 398 (3.97%) were dermatophytes. *Microsporum* species was the most common dermatophyte accounting for 50.5% (*n* = 201) followed by trichophyton with 36.9% (*n* = 147). The most common positive samples were scrapping (251, 63%) and hair (68, 17%). Culture positivity relative to the age groups revealed a cluster of positive dermatophyte species in children < 10 years of age with 215 (54%) of all cases and among 10–19 years of age with 60 (15) of the cases (*p* < 0.001). *Microsporum* species were the prevalent dermatophytes in patients  < 10 years of age, while *Epidermophyton* species were the most frequent dermatophyte species in age groups 10–19, 20–29, and 30–39 years. However, *Trichophyton* species were the most frequent dermatophyte species in individuals 70–79 years. The percentage of *Microsporum* and *Trichophyton* species decreased significantly over time (*p* < 0.001). In addition, there was a significant seasonal variation in relation to *Trichophyton* species. A comparison between the most frequent species showed that there was no difference in relation to gender, but there was a difference in relation to the specimen type and age group.

**Conclusion:**

Dermatophytosis was common among children and adolescent with the most common samples were scrapping and hair. There was a significant reduction in *Microsporum* and *Trichophyton* species over time.

## Introduction

Dermatophytes are saprophytic in nature; however, few dermatophytes have adopted to living on human tissues and may cause serious infections in immunocompromised hosts [1]. Dermatophytes are known to cause superficial mycosis in animals and humans owing to their ability to destroy keratin present in skin, hair, and nails, leading to the development of dermatophytosis. Dermatophytes are categorized into three groups, anthropophilic, zoophilic, and geophilic. The global distribution of dermatophytes is based on this classification and not on the natural habitat and host preferences [2]. Dermatophyte infections may spread either by direct contact with infected people (anthropophilic organisms) or animals (zoophilic organisms), or from contaminated soil (geophilic organisms) [2, 3]. Over 40 species and three significant genera, *Microsporum, Trichophyton*, and *Epidermophyton* are known to cause dermatophytosis in humans [3, 4]. Dermatophytosis, otherwise known as Tinea infections, are prevalent worldwide, but are more common in the tropics due to high level of humidity, overpopulation, and poor hygiene [2]. The transmissibility of the causative agents of dermatophytosis among the human population is variable with Tinea capitis being the most contagious and tineae (corporis, manuum, and cruris) being the least transmissible [5, 6].

One of the dermatophytosis is Ringworm, Tinea Corporis, infection and is known to affect approximately 20–25% of the global population [7]. Dermatophytes represent the majority (90%) of fungal nail diseases (onychomycosis) in the United States and Europe [8]. Previous studies showed that the pervasiveness of dermatophytosis among cutaneous wounds ranges from 18.2 to 23.2% in Brazil [9, 10]. In Nigeria, the prevalence of superficial fungal infections has been reported to range from 3.4 to 55% [11].

In Saudi Arabia, dermatophyte infections are thought to be fairly common [12]. However, only a few studies have been conducted to determine the prevalence and incidence of dermatophyte infections in the country. The first study in Saudi Arabia investigated the presence of fungi in 4294 clinical samples [13]. Among these samples, 680 (15.8%) were tinea pedis and tinea manuum. The causative agents responsible for most (88.9%) of these infections were *Candida* species and other types of yeasts. However, only 11.1% of the infections were caused by dermatophytes [13]. Since dermatophytes are common disease and the distribution of the different species and different age groups might vary overtime, we undertook this study to highlight the prevalence of dermatophytes in a hospital in Eastern Saudi Arabia and evaluated seasonality for most common dermatophyte species.

## Materials and Methods

This study was conducted at the Johns Hopkins Aramco Healthcare (JHAH) in Dhahran, Eastern Province of Saudi Arabia. The JHAH hospital in Dhahran is a 350-bed general hospital and provides medical care for about 160,000 individuals eligible for medical care. The study was approved by the Institutional Review Board (IRB) of Imam Abdulrahman Bin Faisal University (IRB-PGS-2018-03-175) and the IRB of JHAH (IRB #18-21). Fungal culture data from 2000 to 2019 period were obtained from the Laboratory Information System of the Mycology Laboratory at JHAH and from the electronic laboratory information system. We retrospective analyzed all fungal culture results from 2000 to 2019.

### Processing of Clinical Samples

Different types of samples were received, including respiratory samples, blood, purulent materials, nail, hair, skin, aspirates, and body fluids. Using a sterile applicator stick, a section/aliquot of the test samples was removed and placed at the center of a plate containing Sabouraud dextrose agar (SDA) and mycological agar, and then incubated at 27–30 °C in an incubator. All cultures were examined once a week for a total of four weeks. All samples were examined for macroscopic growth-related features, including rapid or slow growing colonies; colony shape (flat, heaped regularly, or irregularly folded); creamy, powdery, granular, or velvety texture; pigmentation; and aspects of the reverse side of the plate (whether it was similar or not).

Any unexpected or unusual growth patterns were examined microscopically using a flamed firm needle after removing a small section of the colony from the most granular area. Then, the mold was placed in a drop of lactophenol cotton blue. A cover glass was then pressed gently over the slide, and the slide was examined under a microscope using lowered light, at low power or high power for the presence of specialized hyphal structures and conidia. The Vitek^®^ MS microbial identification system (biomerieux) was used if the culture was not identified microscopically.

### Statistical Analyses

The data were analyzed using Microsoft Excel and IBM SPSS Statistics 26. Descriptive statistics were used for all continuous and count variables. Frequency tables were presented for categorical variables. Statistical testing was performed using parametric and nonparametric tests based on the variable properties. Independent sample t test and Mann–Whitney *U* test were used for comparing two groups, and Kruskal–Wallis *H* test and analysis of variance (ANOVA) were used for comparing more than two groups. A sequence chart (line graph) was used for the time series representation of the data. A *p* value < 0.05 was considered statistically significant.

## Results

**Fig. 1:**
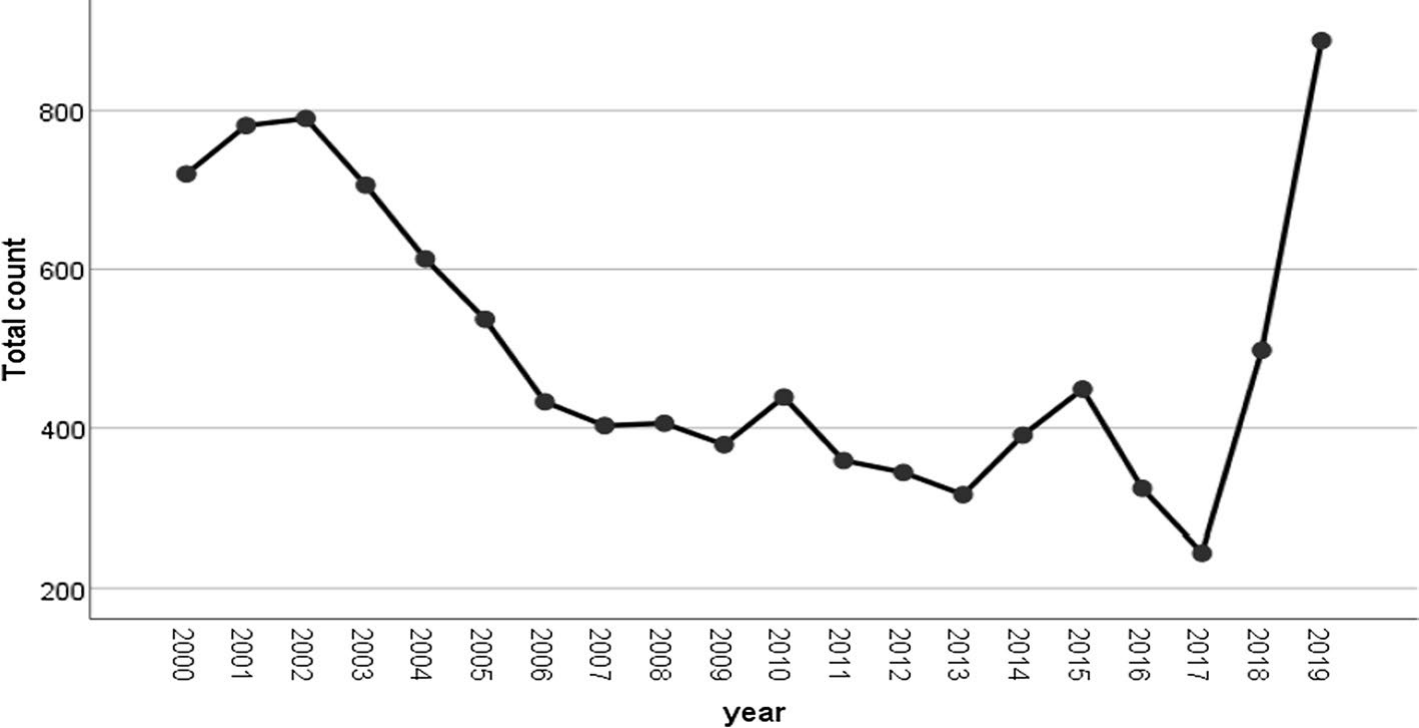
Line chart of yearly number of positive dermatophytes 2000–2019

There was a total of 10,021 fungal cultures during the 20-year study period. The highest number of samples was obtained in 2019, while the lowest number of samples was obtained in 2017 (Fig. 1). The mean age of the patients (± SD) was 37. 63 ± 24.9 years, and 19.2% was children (< 10 years old). The received samples included skin scraping (18%), body swabs (17.6%), nails (17.2%), body fluids (9.2%), tissues (6.7%), aspirates (5.5%), bronchial lavage (5.5%), hair (4.1%), sputum (3.8%), lung wash (3.3%), blood (3.2%), abscess (0.8%), urine (0.6%), and others (4.5%). Of the samples, 5001 (49.9%) were males and 5020 (50.1%) were females. Of all the samples, 3040 (30.33%) were positive for fungi, 2642 (26.36%) were nondermatophytes, and only 398 (3.9%) were dermatophytes.

**Fig. 2:**
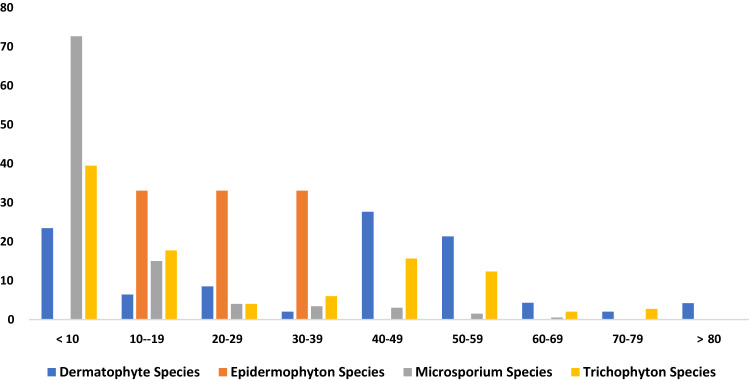
Proportions of the different dermatophytes among different age categories

The details of the analysis of the 398 dermatophytes isolates are provided further. Culture positivity relative to the age groups revealed a cluster of positive dermatophyte species in children (< 10 years; *p* < 0.001) (Table 1). *Microsporum* species were the prevalent dermatophyte species in patients  < 10 years of age, while *Epidermophyton* species were the most frequent dermatophyte species in age groups 10–19, 20–29, and 30–39 years. However, individuals in the age group 70–79 years had *Trichophyton* as the most frequent dermatophyte species (Fig. 2).

**Table 1:** Positivity rate of dermatophyte species according to the age group

Age group	Epidermophyton species	Microsporium species	Trichophyton species	Other dermatophytes	Total
< 10	0 (0)	146 (67.9)	58 (27)	11 (5.1)	215
10–19	1 (1.7)	30 (50)	26 (43.3)	3 (5)	60
20–29	1 (5.3)	8 (42)	6 (31.6)	4 (21)	19
30–39	1 (5.6)	7 (38.9)	9 (50)	1 (5.6)	18
40–49	0 (0)	6 (14.3)	23 (54.8)	13 (31)	42
50–59	0 (0)	3 (9.7)	18 (58.1)	10 (32.2)	31
60–69	0 (0)	1 (16.7)	3 (50)	2 (33.3)	6
70–79	0 (0)	0 (0)	4 (80)	1 (20)	5
≥ 80	0 (0)	0 (0)	0 (0)	2 (100)	2
All age group	3 (0.7)	201 (50.5)	147 (37)	47 (11.8)	398

*Χ*^2^ = 123.85; *p* < 0.001

**Fig. 3:**
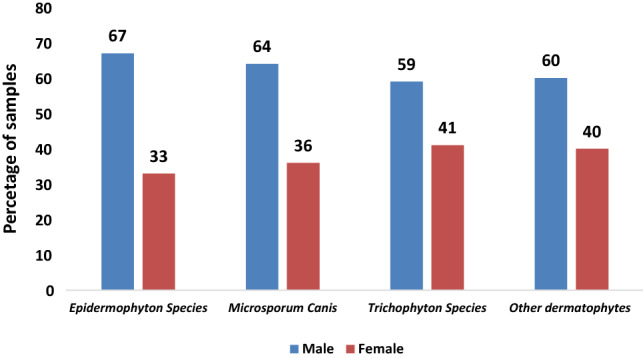
Percentage of dermatophyte species in males and females

**Fig. 4:**
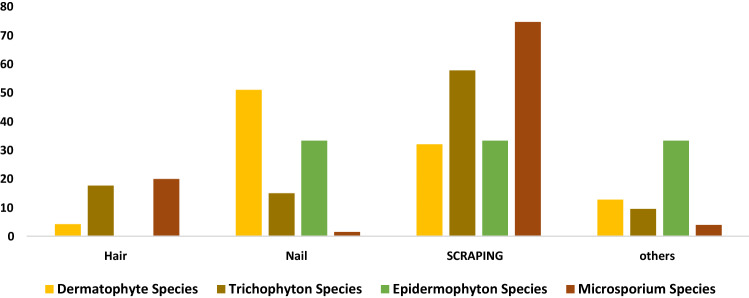
Percent of the distribution of dermatophyte species in clinical samples

Of the 398 dermatophytes, there were 201 (50%) *Microsporium Species*, 147 (36.9%) *Trichophyton species*, 3 (0.75%) *Epidermophyton Species*, and 47 (11.8%) other dermatophyte species. However, the most common organisms were *Microsporum canis* (143, 35.9%), and *Trichophyton violaceum* (58, 14.5%). The most common samples were as follow: skin scrapings (63.6%), hair (17.1%), and nails (12.8%) (*Χ*^2^ = 104.083; *p* < 0.001) (Table 2). *Microsporum* was the most common dermatophyte species isolated from male patients (*p* = 0.04) (Table 3). From all isolated dermatophytes, male patients contributed more to the positivity of isolates (Fig. 3). Skin scraping had contributed the most to the most common dermatophytes (Fig. 4). However, a comparison between the three most frequent species showed that was no difference in gender but there was a difference in the specimen type and age groups (Table 3).

**Table 2:** Positivity rate of dermatophytes in different clinical samples

Specimen type	Epidermophyton species	Microsporium species	Trichophyton species	Other dermatophytes	Total
Hair	0 (0)	40 (58.8)	26 (38.2)	2 (2.9)	68
Nail	1 (2)	3 (6)	22 (44)	24 (48)	50
Scraping	1 (0.4)	150 (59.8)	85 (33.9)	15 (6)	251
Others	1 (3.4)	8 (27.6)	14 (48.3)	6 (20.7)	29
Total	3 (0.75)	201 (50.3)	147 (36.9)	47 (11.8)	398

*Χ*^2^ = 104.083; *p* < 0.001

**Table 3:** A comparison between the different types of dermatophytes species

	Number	Mean age ± SD	Male, *N* (%)	Most common specimen type, (*N*; %)
Dermatophyte species	47	36.596 ± 23.67	32 (68)	Nail (24, 51%)
Epidermophyton species	3	25 ± 11.53	2 (66.7)	Nail, scraping, others (33% each)
Microsporium species	201	9.826 ± 11.64	129 (64.2)	Scrapping (150; 74.6%)
Trichophyton species	147	24.245 ± 21.72	88 (59.9)	Scrapping (85; 57.8%)
*p* value		< 0.001	0.734	< 0.001

**Fig. 5:**
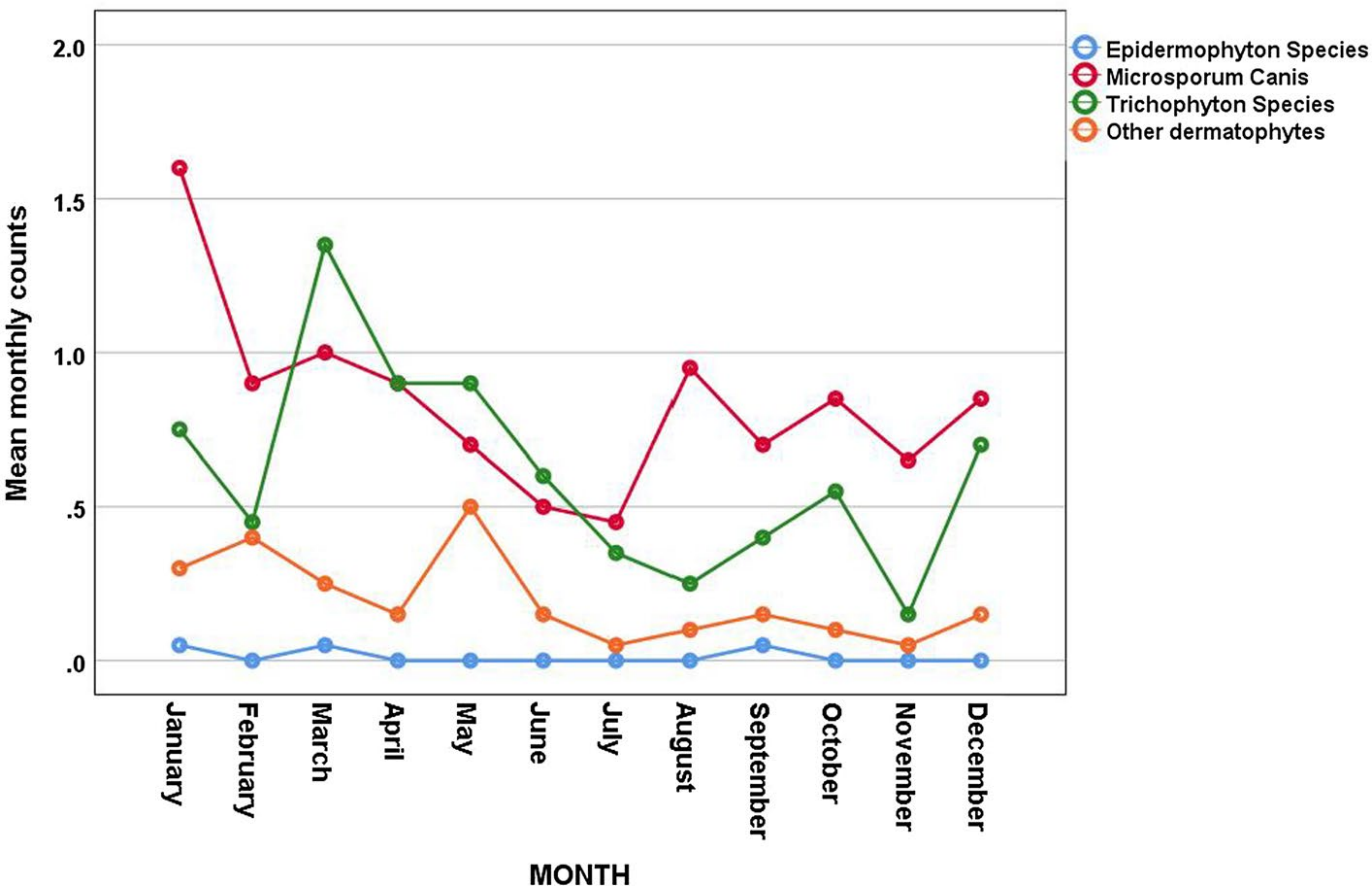
Seasonality of dermatophytes species

The trend of the dermatophyte species was tested using Poisson Harmonic regression analysis. The data showed *Microsporum* and *Trichophyton* species decreased significantly over time (*p* < 0.001 and 0.001, respectively). There was a significant seasonal variation was found in *Trichophyton* species alone (Fig. 5).

## Discussion

Dermatophytes are a group of fungi that may cause cutaneous mycoses and mainly infect superficial keratinized tissues such as skin, hair, and nails [2, 3]. Cutaneous infections have increased in the last decade [7] and 20–25% of the fungal infections worldwide are caused by dermatophytes [2]. In 1993, a study was conducted in Qatif Central Hospital, Saudi Arabia, to determine the prevalence of tinea capitis among Saudi nationals. Clinical samples were collected from 372 patients, and only 240 (64.5%) samples were identified as being positive via direct microscopic examination. The results showed that tinea capitis represents 47.7% of the superficial mycosis infections [14]. The most common etiological agent was *Microsporum canis* (82.3%), followed by *Trichophyton violaceum* (13.9%) and *M. audouini* (2.2%) [14]. *T. mentagrophytes*, T*. rubrum, T. verrucosum*, and *T. simii* were isolated from one patient each [14]. However, the current study showed that the most common organisms were *Microsporum canis* (143, 35.9%), and *Trichophyton violaceum* (58, 14.5%).

Humidity and high temperatures affect the occurrence of dermatophyte infections [15]. In the summer, countries in the Arabian Gulf experience a hot and moist climate. Therefore, dermatophytosis-like tinea corporis and tinea cruris were found to be most common in the Eastern Province of Saudi Arabia [13]. However, another study revealed tinea capitis and tinea pedis to be the most common, and tinea corporis as the least common in the central (Riyadh) region of Saudi Arabia [12]. This difference might be due to differences in environmental conditions. The eastern region extends along the Arabian Gulf, in contrast with the central region, which has a very dry climate. In this study, *Microsporum* species were mostly isolated from the age group of less than 10 years old, similar to previously reported studies from Saudi Arabia [12, 13].

In this study, the most common organisms were *Microsporum canis* (143, 35.9%), and *Trichophyton violaceum* (58, 14.5%). In a recent study from Iran utilizing sequencing methodology showed that the most frequent dermatophytes were *Trichophyton mentagrophytes* (20%), followed by *Trichophyton tonsurans* (10%), *Trichophyton rubrum* (6.7%), *T. interdigital* (6.7%) [16]. In another study from Kuwait, the most common dermatophytes were *Trichophyton mentagrophytes* (39%), *Microsporum canis* (16%), *Trichophyton rubrum* (10%), and *Epidermophyton floccosum* (6.2%) [17]. An additional recent study showed that in diabetic patients in Kuwait, dermatophytes were the most common cause of onychomycosis [18]. In a systematic review of dermatophytes in Brazil, *Trichophyton rubrum*, *T. interdigitale*, and *T. mentagrophytes* were the most common species [19]. A study from Ethiopia showed that *Trichophyton* spp. (32%) *Epidermophyton* spp. (20.2%), and *Aspergillus fumigatus* (8.3%) were the most common dermatophytes [20]. Thus, a wide variation in the organisms of dermatophytes exists between countries and with regions of any country.

In our study, we found that males contributed more to the samples of the dermatophytes than females with no significant difference among both genders in relation to the most frequent isolates. However, there was no difference in the types of dermatophytes among the different age groups. A population-based study from Iceland identified a higher prevalence of dermatophytes among male patients [21] and in India dermatophytosis was associated with a male to female ratio of 1.7:1 [22]. In a recent study from Egypt, male predominance was also noted [4]. This gender difference was attributed to progesterone as it is thought to play a major role in preventing dermatophyte multiplication in vitro [23]. However, in contrast with our findings, one study from Saudi Arabia reported that dermatophyte infections was almost twofold higher in females than that in males [24]. In a study conducted on patients attending a tertiary hospital in Ethiopia, concluded that more females were affected by dermatophytes than males, with a female-to-male ratio of 2.2:1 [2]. These differences are likely related to the pattern of exposure to dermatophytes in the different populations.

In this study, *Microsporum* species, mostly zoophilic dermatophytes, were the most prevalent among dermatophytes isolated in our region. These results are in agreement with previous studies in Saudi Arabia [25, 26]. However, other studies from the Middle East showed tinea to be the most common isolated organisms. In a study from Kuwait of 2730 patients showed the most common organism were *T. mentagrophytes* (39%), *M. canis* (16%), and *Epidermophyton floccosum* (6.2%) [17] and another study showed that the most common organisms were *T. capitis* (71·1%), *Microsprum canis* (60·7%), and tinea capitis (76%) [27]. A study from Lebanon showed that the most common dermatophytes were *Trichophyton* spp. (89.9%) and *Microsporum* spp. (9.1%) [28], and another study from Iran showed the most common organism were tinea pedis (43.4%), tinea unguium (21.3%), and tinea cruris (20.7%) [29]. One study from Turkey showed the predominant organisms to be *Trichophyton rubrum* (62.2%), and *T. mentagrophytes* (16.9%) [30]. In one study from India, *Trichophyton* species were found to be more widely isolated [22]. Researchers worldwide have studied various factors, such as climate, temperature, humidity, personal hygiene, xerosis, age, and socioeconomic factors that affect the presence of dermatophytes in humans [23]. However, the seasonality of dermatophytes over the years has not been studied before in our region. This study found a significant seasonal variation for *Trichophyton* species over the 20-year period, which is agreement with the results of other studies conducted in Korea [31].

Our results showed that *Microsporum* and *Trichophyton* species decreased over time with *p* < 0.001 and 0.001, respectively. The peak for dermatophyte species was reported from December to May (during winter and spring), there was a high prevalence of dermatophyte infections. Whether this result might be related to climate change as a result of increased global warming is unclear. This finding contradicts studies published in India, where the peak incidence of dermatophyte infections was higher during summer months [22]. Interestingly, a study found an increased rate of dermatophyte carriage by cats during winter and spring [32]. However, no similar studies have been performed in the Gulf Cooperation Council countries addressing the association between climate change and the incidence of dermatophytosis.

It is important to track the epidemiology and the burden of fungal infections in general and dermatophytosis in specific. Such activities would help in elucidating the causative agents to develop strategies for prevention and therapy. In addition, there is a need to have more studies to address the interaction of risk factors, such as xerosis, lifestyle, global warming, migration of laborers, synthetic clothing, obesity, and living with pets and the occurrence of dermatophytosis [23]. The precise characterization of dermatophytes is needed to identify the organism to the species level, but this is a common limitation in most of the diagnostic mycology laboratories in our region, probably due to the complex requirements and the tedious techniques involved in full species identification. This limitation may be overcome using more sensitive techniques, such as molecular characterization of dermatophytes and the use of internal transcribed spacer (ITS) sequencing.

In conclusion, dermatophytosis was common among children and adolescent with the most common samples being scrapping and hair. There was a significant reduction of *Microsporum* and *Trichophyton* species over time.

## Declarations

### Data availability statements

Data available upon reasonable request.
